# Insights from Explainable Artificial Intelligence of Pollution and Socioeconomic Influences for Respiratory Cancer Mortality in Italy

**DOI:** 10.3390/jpm14040430

**Published:** 2024-04-18

**Authors:** Donato Romano, Pierfrancesco Novielli, Domenico Diacono, Roberto Cilli, Ester Pantaleo, Nicola Amoroso, Loredana Bellantuono, Alfonso Monaco, Roberto Bellotti, Sabina Tangaro

**Affiliations:** 1 Dipartimento di Scienze del Suolo, della Pianta e degli Alimenti, Università degli Studi di Bari Aldo Moro, 70126 Bari, Italy; donato.romano@uniba.it (D.R.); pierfrancesco.novielli@uniba.it (P.N.); 2 Istituto Nazionale di Fisica Nucleare, Sezione di Bari, 70126 Bari, Italy; domenico.diacono@ba.infn.it (D.D.); roberto.cilli@uniba.it (R.C.); ester.pantaleo@uniba.it (E.P.); nicola.amoroso@uniba.it (N.A.); loredana.bellantuono@uniba.it (L.B.); alfonso.monaco@uniba.it (A.M.); roberto.bellotti@uniba.it (R.B.); 3 Dipartimento Interateneo di Fisica “M. Merlin”, Università degli Studi di Bari Aldo Moro, 70126 Bari, Italy; 4 Dipartimento di Farmacia Scienze del Farmaco, Università degli Studi di Bari Aldo Moro, 70126 Bari, Italy; 5 Dipartimento di Biomedicina Traslazionale e Neuroscienze, Università degli Studi di Bari Aldo Moro, 70126 Bari, Italy

**Keywords:** explainable artificial intelligence, machine learning, remote sensing, air pollution, exposome, respiratory disease, socioeconomic indices, public health

## Abstract

Respiratory malignancies, encompassing cancers affecting the lungs, the trachea, and the bronchi, pose a significant and dynamic public health challenge. Given that air pollution stands as a significant contributor to the onset of these ailments, discerning the most detrimental agents becomes imperative for crafting policies aimed at mitigating exposure. This study advocates for the utilization of explainable artificial intelligence (XAI) methodologies, leveraging remote sensing data, to ascertain the primary influencers on the prediction of standard mortality rates (SMRs) attributable to respiratory cancer across Italian provinces, utilizing both environmental and socioeconomic data. By scrutinizing thirteen distinct machine learning algorithms, we endeavor to pinpoint the most accurate model for categorizing Italian provinces as either above or below the national average SMR value for respiratory cancer. Furthermore, employing XAI techniques, we delineate the salient factors crucial in predicting the two classes of SMR. Through our machine learning scrutiny, we illuminate the environmental and socioeconomic factors pertinent to mortality in this disease category, thereby offering a roadmap for prioritizing interventions aimed at mitigating risk factors.

## 1. Introduction

Success in mapping the human genome has stimulated the complementary concept of the *exposome,* i.e., the measure of the complete environmental exposure of an individual or a population to their surrounding environment and the study of how those exposures relate to health [1]. Research on the exposome in the context of noncommunicable diseases (NCDs), or diseases that cannot be transferred from one person to the other, is relatively novel and advancing impressively [2]. Although NCDs are associated with a genetic predisposition (the genome), exposure to health-affecting environmental parameters has a strong impact on their risk. With the use of Earth observation data, recent research studies in this field are exploring possible associations of NCDs with environmental parameters to identify relevant factors and prioritize intervention strategies to mitigate their effects, in line with one of the targets of the 2030 United Nations Agenda for Sustainable Development [3], i.e., reducing premature mortality from NCDs.

Among NCDs, respiratory tract cancers, which include tracheal, bronchus, and lung cancer, are the leading causes of cancer death [4], registering the highest age-standardized mortality rate among all cancers [5]. Studies focusing on respiratory diseases and/or cancer using Earth observation data include factors such as air pollution, atmospheric factors, and land surface data in their analysis. They recognize air pollution parameters as important factors, such as NO_2_ and O_3_, and identify prominent associations with parameters such as PM 2.5 exposure, asbestos exposure, UV irradiation, and light pollution [6,7,8,9,10,11,12].

In our analysis, we use air quality estimates by the CAMS reanalysis dataset of atmospheric composition [13] and spatial census data at provincial resolution (environmental and socioeconomic variables) to predict standard cancer mortality rates (SMRs) attributable to respiratory tract cancers as provided by the Italian National Institute of Statistics (ISTAT). More specifically, using data from 2019, we estimate the national average SMR and define a binary variable with a value of 1 for provinces with an SMR higher than the average and 0 otherwise. We then use Earth observation and census data to predict this binary variable, comparing the performance of thirteen different models. Finally, we apply an explainable artificial intelligence strategy, thus providing model-agnostic insights into how the best model calculates predictions with the aim of facilitating the determination of feature importance and offering a roadmap for prioritizing interventions [14,15]. Our approach is therefore aimed at contributing to a comprehensive understanding of the relationships between the environment and human health for the specific disease under consideration.

This paper is structured as follows. In Section 2, we describe the input data and the adopted methodologies. More specifically, in Section 2.1, we define our input factors; in Section 2.2, we define our output binary variable; in Section 2.4, we reduce the features of the dataset through a multicollinearity analysis; in Section 2.5, we introduce the 13 machine algorithms and the evaluation metrics used; in Section 2.6, we describe the explainable artificial intelligence approach adopted. In Section 3, we present the results of our analysis that we discuss in Section 4. Finally, in Section 6, we draw our conclusions.

## 2. Materials and Methods

### 2.1. Pollutants Data and Socioeconomic Indices

The Copernicus Atmosphere Monitoring Service (https://ads.atmosphere.copernicus.eu/#!/search?text=&type=dataset&keywords=((%20%22Product%20type:%20Reanalysis%22%20)%20AND%20(%20%22Variable%20domain:%20Atmosphere%20(composition)%22%20)%20AND%20(%20%22Spatial%20coverage:%20Europe%22%20)%20AND%20(%20%22Temporal%20coverage:%20Past%22%20) (accessed on 10 April 2024) (CAMS) platform managed by the European Union’s Copernicus Program provides information on air pollution and air composition globally. It utilizes data from various sources such as satellites, aircraft, ground stations, and numerical models to monitor and analyze real-time air quality and provide forecasts. The pollutants data provided by the CAMS platform are the result of an ensemble median over 9 numerical air quality models [13,16]: CHIMERE (CH) from INERIS (France) [17], EMEP (EM) from MET Norway (Norway) [18], EURAD-IM (EU) from Jülich IEK (Germany) [19], LOTOS-EUROS (LO) from KNMI and TNO (the Netherlands) [20], MATCH (MA) from SMHI (Sweden) [21], MOCAGE (MO) from Meteo-France (France) [22], SILAM (S) from FMI (Finland) [23], DEHM (DE) from Aarhus University (Denmark) [24], and GEM-AQ (GE) from IEP-NRI (Poland) [25]. For the purposes of the present study, which aims at investigating the association between pollution and mortality for cancer with the respiratory system, only the concentrations of CO, NO, NO_2_, O_3_, PM 10, PM 2.5, and SO_2_ were considered. Since the data downloaded from the CAMS platform covered an area larger than that of our interest, the Python library for analyzing geolocalized data https://geopandas.org/en/stable/ (accessed on 10 April 2024) Geopandas (version 0.14.1) was used to extract the pollution data of the Italian peninsula. From the daily coverage over all of 2019, we retrieved the mean values for the concentrations of the selected pollutants.

In order to maximize data consistency, other pollution-related and socioeconomic variables were considered: cultivated areas, urban areas, benzene, temperature, N fertilizer, P4010 fertilizer, microelement fertilizer, organic fertilizer, the number of hospital beds available (bed number), lifetime, income, life quality, instruction, vehicles per km^2^ of land area (vehicles total), vehicles per km^2^ of urbanized surface (urban traffic), photovoltaic panel, percentage of urban greenery density on the surface (green urban), electric consumption, exceeding the limits detected following noise checks (noise), and municipal waste collection in tons (wastes) [26].

### 2.2. Standardized Mortality Ratio

The Italian National Institute of Statistics (ISTAT) is the main official statistical institute in Italy, responsible for collecting, analyzing, and disseminating statistical information about the country.

**Figure 1:**
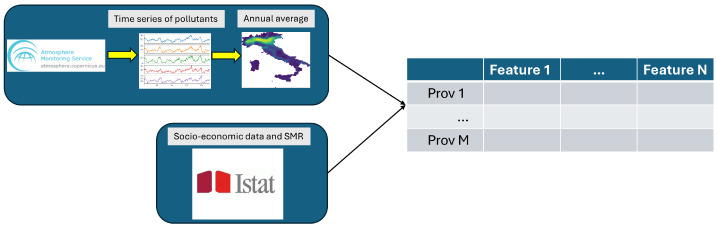
Dataset construction workflow.

**Figure 2:**
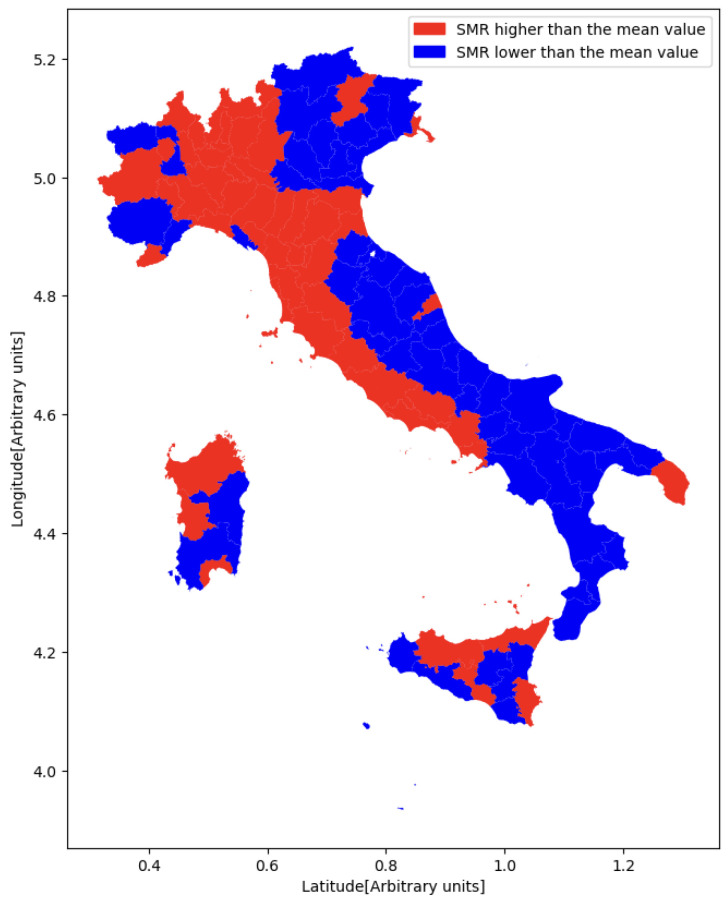
Colormap of the Italian provinces with higher or lower SMR than the mean value for cancer to the respiratory system.

The standardized mortality ratio (SMR) is a measure of the mortality rate of a specific population compared with a standard or reference population (e.g., the national population). It is calculated by dividing the number of observed deaths in the study population by the number of expected deaths based on the mortality rates of the standard population. In our study, the values of the SMR of the Italian provinces were calculated starting from the number of observed deaths for respiratory system cancer in 2019 by age class provided by ISTAT. The mean value of the SMR was used to divide the Italian provinces into two classes: all the provinces with an SMR lower than the mean value were assigned to class 0, while all the others were assigned to class 1. An outline of the data preparation workflow is presented in Figure 1. A graphical representation of Italian provinces with a higher or lower SMR than the mean value for cancer to the respiratory system is shown in Figure 2.

### 2.3. Analysis Flowchart

**Figure 3:**
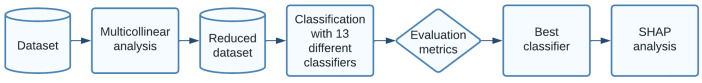
Dataset analysis workflow.

The diagram presented in Figure 3 serves as a visual representation encapsulating the comprehensive analytical journey delineated within this paper. At the heart of this endeavor lies the curation of a dataset, meticulously constructed to encompass a rich tapestry of data streams. The foundational pillars of this dataset were erected through a data acquisition process, commencing with the extraction of air pollution metrics from diverse provinces across the Italian peninsula. These statistics were sourced from the Copernicus Atmosphere Monitoring Service (CAMS) platform, renowned for its robust and comprehensive environmental monitoring capabilities. In tandem with air quality metrics, a plethora of socioeconomic indicators were judiciously incorporated into the dataset. These encompassed a spectrum of socioeconomic variables, ranging from income distribution patterns to urbanization indices, providing a holistic perspective on the societal fabric under scrutiny. Furthermore, the dataset was augmented with mortality data attributable to respiratory system cancers, gleaned from the extensive repository maintained by the Italian National Institute of Statistics (ISTAT). The analysis commenced with the exploration of multicollinearity dynamics inherent within the dataset. Leveraging the Variation Inflation Factor (VIF) as a diagnostic tool, we dissected intervariable relationships to discern and mitigate the pernicious effects of collinearity. Through a systematic iterative process, variables exhibiting VIF indices surpassing the critical threshold of 10 were pruned from the dataset, thereby ensuring the integrity and robustness of subsequent analyses. Subsequently, the refined dataset served as fertile ground for the cultivation of predictive models leveraging state-of-the-art machine learning techniques. Employing the Python (3.10.12 version) library https://pycaret.org (accessed on 10 April 2024) PyCaret (version 3.3.0) as a conduit, a diverse ensemble of 13 distinct machine learning models was meticulously trained and fine-tuned and tasked with the classification of provinces exhibiting mortality rates from respiratory system cancers either surpassing or falling below the national average. The efficacy of these predictive models was rigorously scrutinized through a comprehensive validation process, employing a 10-fold cross-validation methodology by evaluating the performance obtained from the validation folds. Once the model with the largest ROC AUC was identified, the most important features for the model to classify provinces with higher or lower mortality due to respiratory system cancer were calculated using the explainable artificial intelligence algorithm SHAP.

### 2.4. Feature Correlation Analysis

The Variation Inflation Factor (VIF) is a measure used in regression analysis to assess multicollinearity among independent variables. The VIF provides an indication of the strength of correlation among independent variables, detecting the effect of inflation on the variance in estimated coefficients in the regression model.

In this study, the VIF was calculated to identify and address the issue of multicollinearity within our dataset. An iterative approach was adopted, where the VIF was computed repeatedly, eliminating the variable with the highest VIF at each iteration, provided its value exceeded the threshold of 10. This process effectively reduced multicollinearity in the model, thereby improving the stability of coefficient estimates.

The surviving features are mean NO, cultivated areas, benzene, P4O10 fertilizer, microelement fertilizer, organic fertilizer, income, instruction, vehicles total, urban traffic, green urban, and noise.

### 2.5. Comparison of Classification Models

Thirteen different classification models, Gradient Boosting Classifier (gbc) [27], Light Gradient Boosting Machine (lgb) [28], Random Forest Classifier (rf) [29], Extra Trees Classifier (et) [30], K Neighbors Classifier (knn) [31], Extreme Gradient Boosting (xgb) [32], Linear Discriminant Analysis (lda) [33], Ada Boost Classifier (ada) [34], Decision Tree Classifier (dt) [35], Naive Bayes (nb) [36], Quadratic Discriminant Analysis (qda) [37], Logistic Regression (lr) [38], and Dummy Classifier (dum) [39], a classification model that does not learn anything from the training data but is particularly useful for assessing the performance of more complex models and understanding the difficulty of the classification task, were trained on the whole dataset in a 10-stratified cross-validation.

The performance of our machine learning models was evaluated on each validation set through the following metrics:

- Accuracy:
  (1) $$ACC=\frac{TP+TN}{TP+FP+TN+FN}$$
- AUC ROC: The area under the Receiver Operating Characteristic (ROC) curve;
- Recall:
  (2) $$REC=\frac{TP}{TP+FN}$$
- Precision:
  (3) $$PREC=\frac{TP}{TP+FP}$$
- F1-score:
  (4) $$F1=2\cdot \frac{PREC\;\cdot \;REC}{PREC\;+\;REC}$$
- Kappa:
  (5) $$k=\frac{2\cdot (TP\cdot TN-FN\cdot FP)}{\left(TP+FP)\cdot (TP+FN)\cdot (FN+TN\right)}$$

### 2.6. Explainable Algorithm

We adopted the SHAP algorithm [40,41] to explain the decisions of the Gradient Booster Classifier models on each test sample. It provides insight into the contribution of each feature to the model’s prediction for a specific instance. SHAP values are based on game theory concepts and specifically on Shapley values, which originated in cooperative game theory. One of the key advantages of SHAP is its model-agnostic nature. It can be applied to any machine learning model, whether it is a black-box or white-box model, making it versatile and widely applicable. The measurement of how a feature affects the performance of the classification model on the validation set is computed by including and removing it from the model:

(6) $$\Phi_{j}\left(x\right)=\sum_{F\subseteq S-\{j\}}\frac{|F|!(|S|-|F|-1)!}{|S|!}\left[f_{x}\left(F\cup j\right)-f_{x}\left(F\right)\right]$$

where $\Phi_{j}\left(x\right)$ represents the SHAP value of feature *j* for the prediction of the model *f* given the input *x*, *S* is the set of all features, *F*⊆S−{j} represents all possible subsets of features excluding feature *j*, and $\frac{|F|!(|S|-|F|-1)!}{|S|!}$ is a weight parameter that multiplies all of the permutations of S! by the potential permutations of the remaining class that does not belong to S, while $f_{x}\left(F\cup j\right)$ and $f_{x}\left(F\right)$ denote, respectively, the model’s prediction when feature *j* is added to the subset *F* and when it is absent. We also averaged the ten realizations of SHAP values in order to obtain a single representative SHAP vector. The whole analysis workflow is shown in Figure 3.

## 3. Results

The aim of this study was to assess, using explainable machine learning models, the extent to which air pollutants and socioeconomic descriptors are associated with higher or lower mortality from respiratory system cancer.

### 3.1. Performance of Classification Models

Table 1 shows the average performance of the 13 machine learning classifiers calculated as the mean performance obtained during the 10-fold cross-validation. The classifiers are sorted in descending order according to the accuracy values.

**Figure 4:**
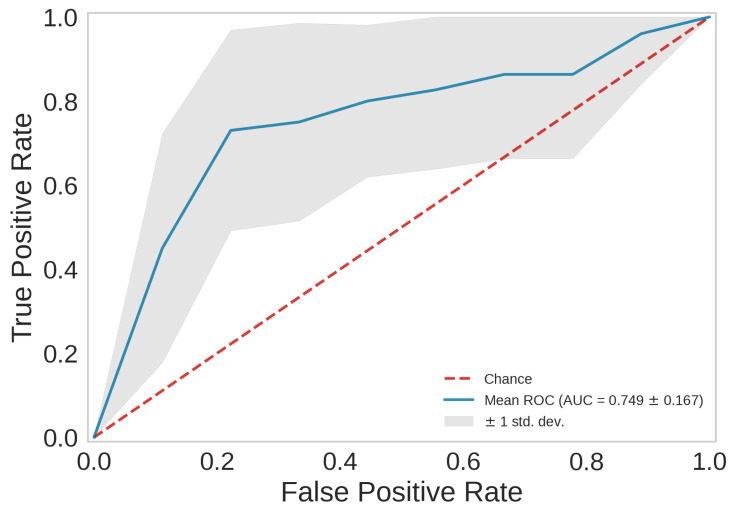
Average ROC curve with standard deviation of the Extra Trees classifier over 10 validation sets.

The Extra Trees classifier emerged as the best in predicting an SMR higher or lower than the mean value of mortality from respiratory system cancer, with a mean accuracy of 0.74 ± 0.13. The mean ROC AUC of the best model is shown in Figure 4. Each fold of the cross-validation process yielded a distinct AUC value, allowing us to compute the mean AUC across all folds and assess the model’s overall performance.

### 3.2. Interpreting Model Predictions: Insights from SHAP Analysis

**Figure 5:**
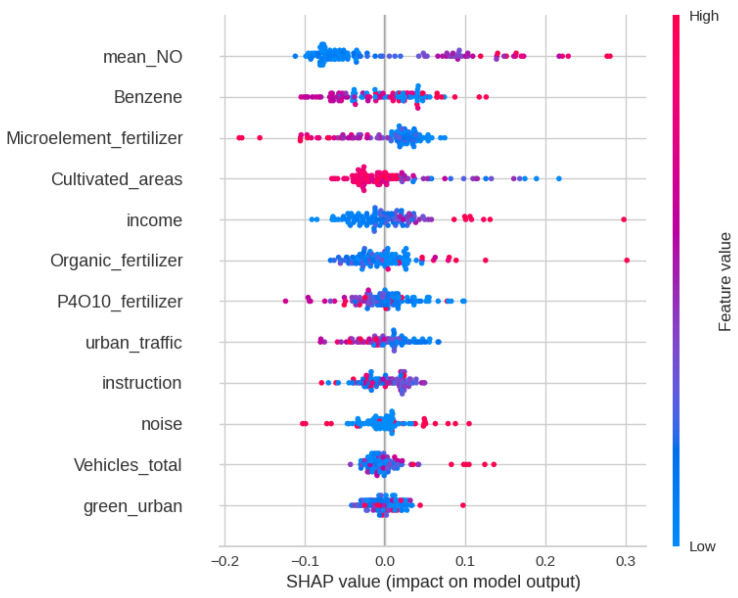
Shap summary plot. It reports the SHAP values, represented along the x-axis, of the dataset features, reported on the y-axis. Each point on the graph represents a province.

In this section, we present a summary plot of the SHAP values obtained from a binary classification model, shedding light on the influential features driving the model’s predictions. Within the 10-fold cross-validation, SHAP values of the features of the provinces present in the validation fold were calculated after training the classification model with the nine folds of training data. This procedure was repeated for each of the validation folds. The summary plot provides a comprehensive overview of the impact of each feature on model predictions, revealing both the direction and magnitude of their influence. Figure 5 illustrates the summary plot generated from the SHAP values computed for the binary classification model. Each point in the summary plot represents a feature, with its position on the y-axis indicating the feature’s importance in terms of absolute SHAP value. The color of each point represents the feature’s value, with red indicating high values and blue indicating low values. Additionally, the horizontal bars represent the impact of each feature on model predictions, with longer bars signifying a stronger influence.

## 4. Discussion

Our findings show that most of the input variables included in this study are weakly correlated to the target variable, the standard mortality ratio, while our Extra Trees model accurately predicts provinces withan SMR higher than the mean value. Therefore, it is likely that a nonlinear relationship between air quality and SMR exists, thus suggesting the beneficial role of including machine learning tools in this analysis. This is also consistent when comparing the classification performance of the tree-based algorithms with other shallow classification algorithms, including the LDA (and others). In ecological studies, linear models should be preferred since they are easy to interpret; however, Extra Trees combined with the SHAP is a robust choice that does not require any prior assumptions, while providing local feature importance values that are completely intelligible.

We also used the distribution of the SHAP values as a tool to estimate the global feature importance of the variables included in our analysis. Accordingly, this analysis confirms that average exposure to NOX is the most important feature in predicting the SMR of respiratory system cancer that is higher than the mean value. This result is in agreement with previous analyses confirming the positive association between respiratory diseases and exposure to NO when conducting studies on populations [42,43].

According to this study, the second most important feature was the average benzene concentration per province. Chronic exposure to benzene has been linked to leukemia [44,45]; however, there is limited evidence of a link with respiratory cancer in the scientific literature [45,46,47]. Nevertheless, benzene pollution is typically generated because of biomass combustion due to wildfires [48] and vehicle exhaust [49]. Hence, we believe this variable is a proxy for population exposure to poor air quality due to smoke from combustion from human and natural activities.

Minor effects can also be imputed to microelement fertilizer consumption and extension of the cultivated areas. As can be seen in Figure 5, the distribution of the related SHAP values suggests that these two variables act as a proxy for living in a rural environment.

Another important feature was the mean income per province. Although it is known that socioeconomic inequalities have a role in respiratory cancer outcomes [50], this effect cannot be assessed through a population study. Besides, it is likely that the average income per province is acting as a proxy for residing in high-populated areas, where most of the air pollution is emitted [42], while not accounting for social disparities.

## 5. Limitations

Key limitations in the proposed workflow stem from the dataset’s limited size and the utilization of standard mortality ratios aggregated at provincial levels to explore associations between respiratory cancer mortality and air pollution. In fact, the dataset’s limited size hampers the ability to discern reliable nonlinear relationships due to the significant influence of outliers and noise. Consequently, we employed a k-fold cross-validation framework and then computed confidence intervals for both predictions and local feature importance. On the other hand, the reliance on aggregated mortality ratios for individual provinces exposes the study to the ecological fallacy, as well as to potential omitted variable bias [51,52], meaning that conclusions drawn at the group level may not accurately represent individual-level associations or causality. Finally, it should be stressed that most of the important variables according to the SHAP algorithm may act as a proxy for the high density of urban activities. If a causality exists, this might be related to the role of NOx in contributing to the development of asthma and respiratory infections, causing a range of harmful effects on lungs [53,54,55]. On the other hand, NO is also related to proximity to residential areas and human activities, so the measured positive association could be partly attributable to other air pollutants, including particulate matter [42]. This work presents a first attempt to extensively evaluate the statistical association between air quality and respiratory cancer mortality over the Italian provinces while accounting for effects from confounding socioeconomic variables. Our conclusions are in agreement with previous cohort studies and meta-analyses [42,56]. The database considered here examined a single year of observations; thus, extending the temporal range of our study could grant increased robustness to the analyses and design a regression approach to model the SMR due to respiratory cancer.

## 6. Conclusions

This paper explores the intricate relationship between air pollution and respiratory cancer, with a focus on cancers of the lung, trachea, and bronchi. Recognizing the urgent public health challenge posed by respiratory cancers, we address the need to identify the most harmful pollutants to inform targeted policy interventions. Our study introduces a novel approach using explainable artificial intelligence based on remote sensing data and socioeconomic data to predict Italian provinces with respiratory system cancer mortality rates higher or lower than the average, which offers several implications for formulating regulations and allocating resources. The results of the research can provide an empirical basis for adopting or strengthening regulations regarding air quality. Identifying provinces with higher mortality rates for respiratory system cancers can highlight areas with higher levels of air pollution and provide an incentive for implementing policies aimed at reducing atmospheric pollution. The ability to predict provinces with respiratory system cancer mortality rates higher or lower than the average can guide more efficient allocation of resources. Areas identified with higher risk may require additional investments in prevention programs, early diagnosis, and treatment of respiratory system cancer, as well as interventions to improve air quality. The adoption of stricter regulations on air pollution and the targeted allocation of healthcare resources can improve the respiratory health of a population and reduce the incidence and mortality of respiratory system cancers.

## Figures and Tables

**Table 1:** Performance of different classification models.

Model	Accuracy	AUC	Recall	Prec.	F1	Kappa
et	0.78 ± 0.19	0.75 ± 0.17	0.73 ± 0.26	0.82 ± 0.18	0.75 ± 0.23	0.57 ± 0.37
lgb	0.74 ± 0.10	0.86 ± 0.13	0.71 ± 0.16	0.80 ± 0.18	0.73 ± 0.10	0.47 ± 0.21
rf	0.72 ± 0.21	0.80 ± 0.16	0.70 ± 0.25	0.78 ± 0.23	0.70 ± 0.22	0.44 ± 0.42
xgb	0.70 ± 0.16	0.78 ± 0.18	0.68 ± 0.24	0.75 ± 0.18	0.67 ± 0.19	0.39 ± 0.32
gbc	0.68 ± 0.18	0.77 ± 0.19	0.68 ± 0.18	0.72 ± 0.21	0.68 ± 0.16	0.36 ± 0.36
ada	0.67 ± 0.28	0.72 ± 0.17	0.68 ± 0.21	0.67 ± 0.17	0.67 ± 0.17	0.34 ± 0.36
qda	0.65 ± 0.12	0.77 ± 0.14	0.50 ± 0.17	0.73 ± 0.23	0.58 ± 0.16	0.30 ± 0.26
lr	0.64 ± 0.13	0.68 ± 0.17	0.56 ± 0.22	0.71 ± 0.20	0.59 ± 0.17	0.28 ± 0.26
knn	0.64 ± 0.16	0.69 ± 0.15	0.58 ± 0.14	0.69 ± 0.21	0.62 ± 0.15	0.27 ± 0.32
lda	0.62 ± 0.13	0.66 ± 0.21	0.50 ± 0.18	0.70 ± 0.22	0.55 ± 0.16	0.23 ± 0.26
dt	0.61 ± 0.21	0.61 ± 0.21	0.61 ± 0.21	0.64 ± 0.22	0.61 ± 0.19	0.21 ± 0.42
nb	0.55 ± 0.08	0.47 ± 0.17	0.17 ± 0.13	0.60 ± 0.44	0.25 ± 0.18	0.09 ± 0.14
dummy	0.50 ± 0.04	0.50 ± 0.00	0.00 ± 0.00	0.00 ± 0.00	0.00 ± 0.00	0.00 ± 0.00

## Data Availability

The data that support the findings of this study are either publicly available on databases cited in the bibliography or available from the corresponding author on request.
